# Does GP empathy influence patient enablement and success in lifestyle change among high risk patients?

**DOI:** 10.1186/s12875-020-01232-8

**Published:** 2020-08-08

**Authors:** Caroline Braad Hansen, Kristian Møller Hornbæk Pavlovic, Jens Sondergaard, Trine Thilsing

**Affiliations:** Research Unit for General Practice, Department of Public Health, University of Southern Denmark, Odense, Denmark

**Keywords:** GP empathy, Lifestyle change, Healthier lifestyle, Empathy

## Abstract

**Background:**

Chronic lifestyle-related-diseases can be prevented by healthy lifestyle. Patients at high risk of disease may benefit from targeted health checks in general practice. However, general-practice-based-studies have shown that patient outcome, enablement, and well-being may be influenced by general practitioner (GP) empathy. The aim of this study is to investigate 1) how high risk patients evaluate their GPs’ empathy during a health check consultation, 2) whether the perceived GP empathy is associated with the patient’s enablement in immediate continuation of the health check consultation and 3) the patient’s subsequent lifestyle changes.

**Methods:**

This study is part of a population based non-randomized feasibility study testing a complex intervention that systematically identifies citizens at high risk of lifestyle-related disease and with health-risk behavior and offers targeted preventive services in the Danish primary care sector. The ultimate aim of the intervention is to improve lifestyle and thereby reduce the risk of lifestyle-related disease. In the feasibility study a random sample of patients aged 30 to 59 years were invited to participate, and to fill in a questionnaire on lifestyle-risk factors. Participants deemed to be at high risk of disease were offered a focused clinical examination and a subsequent health check consultation at the GP. Following each health check consultation GP empathy and patient enablement were assessed using The Care Measure (CARE) and Patient Enablement Instrument (PEI). Patient’s perceived healthy-lifestyle change (y/n) was assessed after three months. The study has been approved by the Danish Data Protection Agency (J.nr 2015–57-0008) and registered at ClinicalTrial. Gov on June 13, 2016.

**Results:**

Twenty-six GP’s participated in the study. Among 93 patients receiving a health check consultation 60 rated the GPs empathy. The median CARE-score was 40. The PEI median was 5.5 and 44.9% achieved a healthier lifestyle. No association was observed between GP empathy and patient enablement or a perceived healthier lifestyle.

**Conclusion:**

No statistical significant association between the CARE-score and patient enablement or a perceived healthier lifestyle was observed. Our results contrast previous findings and may to some extent be explained by a small sample size and the selected high-risk group.

**Trial registration number:**

NCT02797392.

## Background

Lifestyle-related diseases represent a major burden to the healthcare systems world-wide [1, 2]. Most of these diseases can be prevented by lifestyle improvements and disease prevention is a central part of the GP’s work in Denmark and the Nordic countries. Through a specific focus on changing health-risk behaviours and encouraging preventive medical treatment, primary prevention may be able to mitigate the development and impact of chronic diseases. In particular people at high risk of chronic disease may benefit from targeted general-practice-based health checks [3, 4]. As preventive programs often suffer from low or moderate participation rates, the unique doctor-patient relationship in general practice may be important in overcoming such obstacles [5–7]. In addition, a good relationship is important in reaching effective motivational interviewing, which in itself is important to induce behavioural changes in patients [8]. Besides the doctor-patient relationship, the level of GP empathy has been shown to positively affect patient health [8–12].

Although definitions may vary, empathy in a clinical context has been described as an interpretation of the patient’s situation, perspective and feelings, and also the communication of an understanding and willingness to help the patient under his or her conditions [10]. Empathy in a clinical setting is not just an “inner feeling”, but rather a supportive communication which empowers the patient to learn and cope more effectively with the present issues and a reduction or resolution of the patient’s problems [10, 11]. Clinical empathy can be taught [10, 11] and it is a central part of the motivational interview (MI) - a patient-centered interview with the purpose of motivating the patient to change behavior [8].

The impact of GP empathy has been assessed in many different patient groups and clinical settings, and GP empathy has been shown to positively affect patient enablement, various health outcomes and well-being [9, 12, 13]. In addition, GP empathy may have a positive impact on secondary disease prevention, as patients seen by a GP with high empathy has been shown to have better HbA1c- and LDL-cholesterol control [14]. Such results indicate that high GP empathy may facilitate primary prevention (through increased patient enablement) as well as secondary prevention of lifestyle related diseases (through adherence to statins and glucose lowering medication). However, to our knowledge, the specific impact of GP empathy during preventive health checks has not been studied. As previously stated, disease prevention is a central part of the GPs tasks in Denmark and the other Nordic countries, but results from Sweden show that although two out of three GPs would like to prioritize the preventive activities more, variations in the perception of health promotion and prevention, a high work load, lack of guidelines and an unclear purpose hinder a more widespread practice [15–17]. Such barriers and challenges may possibly affect the GPs motivation and empathy during preventive health checks, and in turn affect the short-term (patient enablement) and long-term outcomes (lifestyle changes).

The present study focus on the role of GP empathy during preventive health checks in general practice. The health checks are targeted at high risk patients as this target group may benefit the most from general practice based preventive health checks [3, 4].

The specific aims of this study are to investigate 1) how high risk patients evaluate their GPs’ empathy following a health check consultation, 2) whether the perceived GP empathy is associated with the patients’ enablement in immediate continuation of the health check consultation and 3) the patients’ subsequent lifestyle changes.

## Methods

The TOF pilot study [18] (TOF is a Danish acronym for Early Detection and Prevention) is a population based non-randomized intervention study conducted in the Region of Southern Denmark. It targets adult citizens born between 1957 and 1986 and living in the two Danish municipalities Haderslev and Varde. The aim of the TOF pilot study was to test the acceptability, feasibility, and short-term effects of a step-wise selective preventive program, designed to systematically identify citizens at high risk of lifestyle-related disease and citizens with health-risk behavior and to offer targeted preventive services at the GP and municipality, respectively. The full TOF intervention and characteristics of the participating GPs and municipalities are described in detail elsewhere [6, 18].

**Fig. 1:**
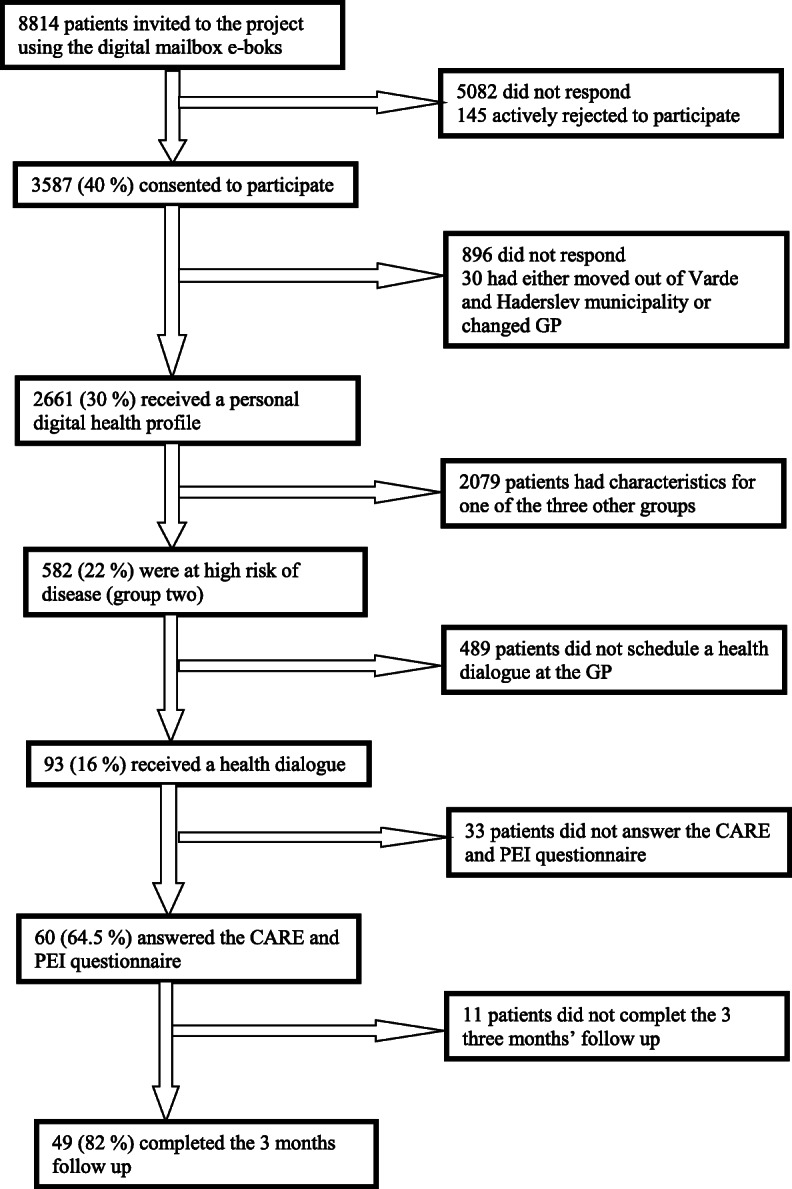
flowdiagram of the selectionproces

A random sample of 8814 patients from 18 participating GP clinics were invited to take part in the study (April 2016) (Fig. 1). The invitation was sent on behalf of the GP and the municipality.

In September 2016 participating patients filled in a questionnaire and were stratified into 4 risk groups based on self-reported information on lifestyle, height, weight and on electronic patient record (EPR) information on diagnoses and treatment of lifestyle-related diseases. Group 1 comprised patients already diagnosed with a lifestyle related disease, Group 2 was patients at high risk of lifestyle-related disease, Group 3 was patients with health-risk behavior and Group 4 was patients with a healthy lifestyle.

The risk of developing a lifestyle-related disease was calculated using validated algorithms for Type 2 Diabetes Mellitus [19], Chronic Obstructive Pulmonary Disease [20] and Cardio-Vascular disease [21].

All risk groups received a digital personal health profile with information about their health and targeted advise on lifestyle changes, if necessary.

This study focuses exclusively on the patients from Group 2. Group 2 patients were at high risk of disease and were therefore offered a targeted intervention at the GP consisting of a focused clinical examination and a subsequent health check consultation. The focused clinical examination comprised measurements of HbA1c, blood cholesterol, height, weight, blood pressure (BP), and, if deemed relevant, lung function measurements and electrocardiogram (ECG). The results were registered in a digital support system with interfaces for both the GP and the patient. After the clinical examination, the patients were invited to prepare for the subsequent health check consultation by answering a questionnaire about their motivation, resources, former experiences with behavioral change, social network, mental health and potential barriers to behavioral change. Based on results from the clinical examination and the questionnaire the GP and patient prepared a health plan including a goal, time frame and identification of the appropriate means to fulfill the plan.

After the health check consultation the patients received an electronic questionnaire with questions on GP empathy and patient enablement. Patient enablement was assessed by The Patient Enablement Instrument (PEI) [22] which contains six questions about enablement and each question holds 0–2 points. This gives a total score from 0 to 12 points with a higher score indicating a better enablement. GP empathy was assessed by The Care Measure (CARE) with 10 questions designed to measure different aspects of clinical encounter related to empathy seen from the patients’ perspective. CARE has been validated in primary care settings [23]. Each question holds a score range from 1 to 6 and a total score of 10–60 points. Before use, the original English version of the CARE Measure was translated into Danish by two native Danish speakers fluent in written and spoken English (an administrator and a secretary at the Research unit for General Practice, Department of Public Health, University of Southern Denmark). The two versions were combined into a draft of the Danish version of the CARE Measure, and the face validity was tested with 10 randomly selected adult patients (5 men, 5 female) from a local GP practice. Based on their feedback, minor amendments were made, to make the items more self-explanatory. The Danish version of the CARE measure was then back translated into English (by a secretary at the Research unit for General Practice). The back-translation was compared to the original CARE measure, and the two versions were found to be very similar.

Three months after receiving the digital personal health profile the participants received an electronic follow up questionnaire including the question “Have you had a healthier lifestyle within the last 3 months?” with answer options “yes” or “no”. All questionnaire answers were kept confidential and were not shared with the GP.

Logistic regression was used to analyze the differences between patients with and without follow-up, and differences in CARE-score between genders was assessed using students t-test. Linear regression was used to assess the association between the CARE-score and the PEI-score. Due to non-normality, the PEI-score was dichotomized around the median and the association between the CARE-score and the PEI-score was re-assessed using logistic regression. Results from these analyses are not shown, as they did not alter the conclusions made based on linear regression.

Logistic regression was used to assess the association between the CARE-score and a healthy lifestyle change at 3 months’ follow-up.

Regression models assessing the difference between patients with and without follow up were adjusted for gender and age, models assessing the association between the CARE-score and the PEI-score were adjusted for gender, age and id of the GP, and models assessing the association between the CARE score and a healthy lifestyle change were adjusted for gender, age, id of the GP and baseline risk behaviours.

All statistical analyses were conducted in STATA version IC16.

## Results

A total of 2661 of the 8814 patients agreed to participate and received a digital personal health profile. Participation rate was higher among women, older patients, patients of higher socioeconomic status, and patients not diagnosed with a lifestyle-related disease [6].

Among the 2661 patients receiving the digital personal health profile a total of 582 were at high risk of developing a lifestyle-related disease. The high risk group was characterized by older age, more men and a lower educational attainment compared to patients with a healthy lifestyle [6]. A total of 93 high risk patients attended the clinical examination and subsequent health check consultation at their GP. More women, patients with sedentary leisure-time behaviour and patients with low self-efficacy attended the health checks [24]. Sixty patients subsequently answered the CARE and PEI questionnaires and 49 also completed the 3 months follow-up. The empathy assessments were performed on 26 GPs from 15 GP clinics. Baseline characteristics for patients with and without follow-up are presented in Table 1.

**Table 1:** Baseline characteristics and patient-assessed GP empathy among patients with and without 3-months’ follow-up

	Patients assessing GP empathy				
	With follow-up (*n* = 49)	Without follow-up (*n* = 11)	P unadjusted	P adjusted*	All (*n* = 60)
Mean age (years, sd)	54.1 (4.0)	53.7 (4.1)	0.39	–	53.9 (4.8)
Male gender (n, (%))	24 (49)	5 (45)	0.83	–	29 (48)
Daily smoker (n,%)	7 (14)	0 (0)	0.18	–	7 (12)
Unhealthy diet (n,%)	14 (29)	3 (27)	0.93	0.75	17 (28)
Sedentary lifestyle (n,%)	13 (27)	1 (9)	0.22	0.23	14 (23)
High risk alcohol intake (n,%)	2 (4)	0 (0)	0.50	–	2 (3)
BMI (median, iqr)	29.4 (5.4)	29.8 (4.8)	0.84	0.70	29.4 (5.3)
CARE score (median, iqr)	40 (8)	36.2 (18)	0.36	0.37	40 (11.4)

*) Analyses adjusted for gender and age

No statistically significant differences were observed between the two groups.

The median CARE-score was 40. There was no significant difference between male and female (*p* = 0.73) nor for age (*p* = 0.65) in the evaluation of the GP empathy.

**Fig. 2:**
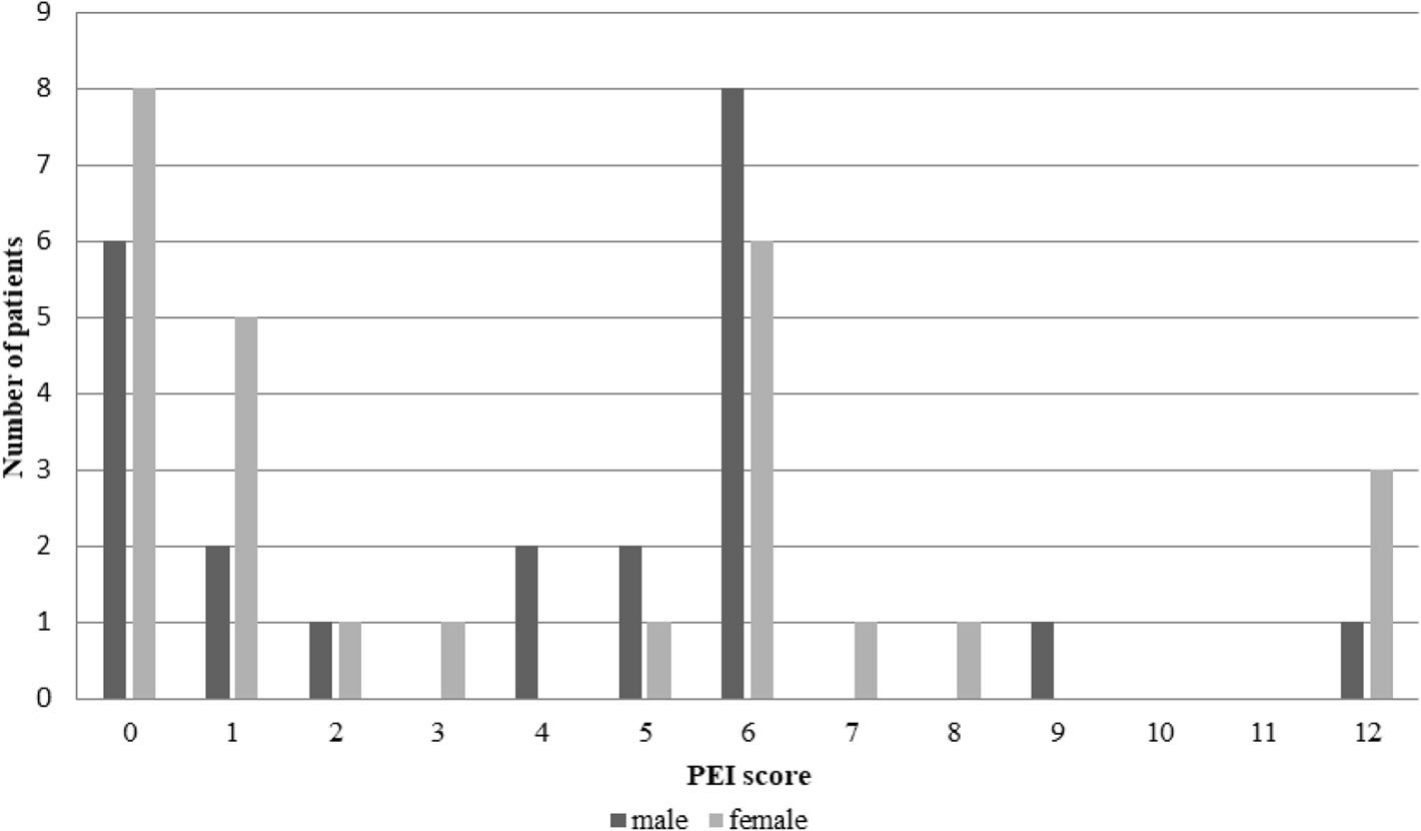
Distribution of Patient enablement instrument (PEI) scores among male and female patients

The median PEI score was 5.5. The PEI score was significantly higher for male patients (median PEI = 6) compared to female patients (median PEI = 3) (Fig. 2).

**Fig. 3:**
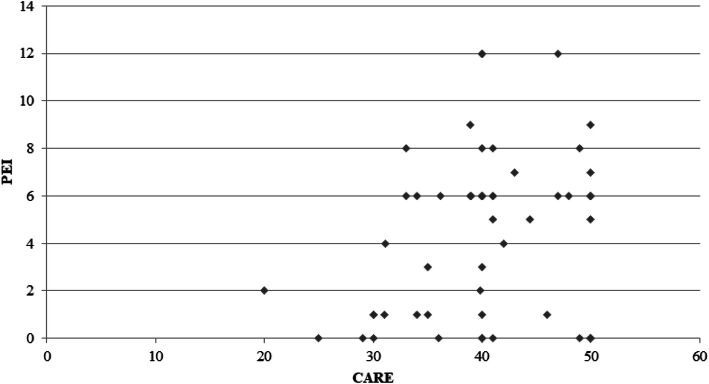
linear regression between CARE-measure score and Patient Enablement Instrument (PEI) score

There was no statistically significant association between the CARE-score and PEI (*p* = 0.132) (Fig. 3), neither when adjusted for age, gender and id of the GP.

**Fig. 4:**
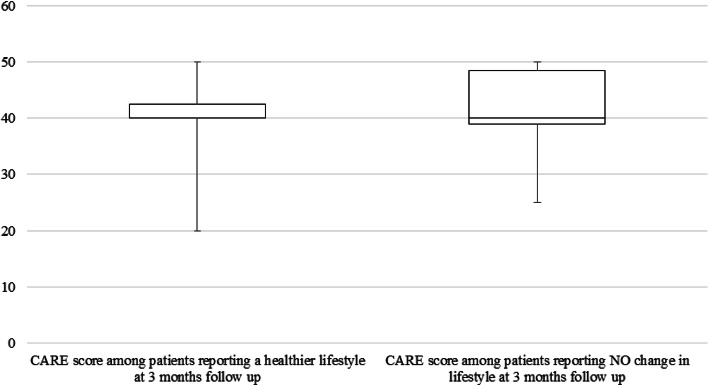
Box-plot of CARE-measure score in patients with and without healthy lifestyle changes at 3 months follow up

At 3 months’ follow-up 44.9% (*n* = 22) of the 49 patients claimed to have achieved a healthier lifestyle within the past 3 months. There was no statistically significant association between CARE-score and a healthy lifestyle change at 3 months follow up (*p* = 0.48) (Fig. 4), neither after adjusting for gender, age, id of the GP and baseline risk behaviors.

## Discussion

The patients assessed GP empathy was relatively high (median CARE score: 40), but surprisingly we did not find any association between GP empathy and patient enablement, nor between GP empathy and healthy lifestyle changes at 3 months’ follow-up.

These results are in contrast to most previous studies showing significant associations between GP empathy and patient enablement [9, 13] as well as between GP empathy and different clinical and lifestyle-related outcomes [9, 13, 14, 25].

Although based on a relatively small group of highly selected patients our results could indicate that GP empathy during health check consultations may not have a great impact on patient’s subsequent lifestyle changes. However, more studies including a broader target group and objective measures of lifestyle changes are needed to confirm the results.

### Limitations and strengths

As indicated, the selected and limited number of participants may introduce selection bias thereby reducing the generalizability of the results. The group of high risk patients attending the health check consultation may well be representative of high risk patients taking up preventive programs in general practice, but they most likely differ from the general high risk patient by having a relatively higher socioeconomic status***.*** In addition, the limited number of participants at follow up (*n* = 49) pose a challenge. Based on simulations with our sample size, the power to detect an odds ratio of 1.5 (associated with a one SD unit increase in CARE score and alpha = 0.05) was about 20%, and about 50% for an odds ratio of 2. Therefore, the probability to detect a difference in CARE score between the patients who claimed to have a healthier lifestyle compared to the ones who did not, was limited.

Lifestyle change was assessed by self-reported measures using a dichotomous variable (yes/no). Such assessment may therefore be subject to reporting bias. However, we have no reasons to believe that such bias would be systematically linked to the assessment of GP empathy.

We used PEI and CARE-measure to evaluate the patient enablement and the GP empathy. Both are validated [22, 23] questionnaires used in previous studies on GP empathy and patient enablement. Furthermore, we used the patients’ assessment of GP empathy which is often more relevant than the GP’s opinion [26].

## Conclusion

High risk patients taking up preventive health checks in general practice generally rated the empathy of their GP as high.

No statistically significant association was seen between GP empathy and the success in getting a healthier lifestyle, neither between GP empathy and patient enablement.

Whether the GP empathy is associated with patient enablement and a healthier lifestyle in high risk patient needs further research. Furthermore, we do believe, that the results should be tested in another setting to achieve more detailed knowledge about the effect of the GP empathy.

## Data Availability

The data that support the findings of this study are not publicly available. Data will however be available from the authors upon reasonable request and with permission from the Danish Data Protection Agency.

## Abbreviation

GP: General practitioner
CARE: The care measure
PEI: Patient enablement instrument
TOF: TOF is a Danish acronym for early detection and prevention
ERP: Electronic patient record
ECG: Electrocardiogram
BP: Blood pressure
